# Bibliographical Notices

**Published:** 1853-09

**Authors:** 


### BIBLIOGRAPHICAL NOTICES.

*On Diseases of the Liver.* By George Budd, M. D., F. R. S., Professor of Medicine in King's College, London, etc. *Second American, from the last and improved London edition. With Coloured Plates and Wood Cuts.* Philadelphia. Blanchard & Lea. 1853. pp. 468.

The lapse of seven years between the publication of the first and second editions of this valuable work, has imposed upon the author the necessity of more than usual revision, modification, and addition; for, certainly, upon no subject within this period, has more new light been thrown, than upon the anatomical structure and functions of the liver, and the chemical composition of its chief secretion.

Dr. Budd, in his voluminous introduction to this edition, presents a lucid and detailed account of the recent discoveries in relation to the intimate structure of the liver, which have led him, in many particulars, to modify his previously expressed opinions on this subject. Among the late observations of anatomists upon which he has drawn, in his minute anatomy of this viscus, we may mention those of our countryman, Dr. Leidy.

As regards the composition of the biliary secretion, the improved analysis on this point since Dr. Budd's first edition have of course led him to very considerable modification of the account which he then gave of the constituents of the bile. After presenting the more recent views of prominent chemists on this point, the author adopts that of Strecker, whose "researches have shown that the biliary matter, which is (as shown by previous chemists) combined with the soda, may be resolved into two acids: both containing nitrogen, and one of them containing sulphur."

The highly important and interesting discoveries of M. Bernard, as regards the formation of diabetic sugar in the blood, during its passage from the liver, receive, we need hardly say, the notice to which they are so well entitled at the hands of Dr. Budd. While admitting, however, the demonstration of M. Bernard, that sugar is formed habitually in the liver, "it seems improbable, he thinks, that the production of a substance which is to be had at so cheap a rate in nature, is an essential office of this organ, at least in herbivorous animals; for not only do these animals find sugar in considerable quantities ready made in their food, but a great part of the starch they consume is also speedily converted into sugar by the action of the salivary and pancreatic juices. It seems more probable that the sugar formed in the liver is incidental and complementary to some more important product."

The subject-proper of the book, the Diseases of the Liver, is illustrated in this edition, by numerous additional observations and cases which have since occurred in the practice of the author. We are unable to notice them verbatim; but we can most unhesitatingly recommend the work as a most complete and learned treatise on this important subject,—a subject of the highest interest to the numerous class of our readers, whose range of practice is within the more southern portions of our country.

---

*Elements of Health, and Principles of Female Hygiene.* By E. J. Tilt, M. D., Physician to the Farrington General Dispensary, &c., &c. Philadelphia. Lindsay and Blakiston. 1853.

Dr. Tilt, in the elegant little volume before us, has done for woman, what Johnson, Combe, Mayo, Thomson, and Southwood Smith had previously done for man, and has presented to the profession, as well as to the general reader, an excellent treatise on the general hygienic treatment of females from infancy to old age. We know not when we have read a book of similar design, which, upon the whole, has pleased us so much as this one. Its principles are sound, philosophical, and intelligible to all. We should be glad to see it in the hands of every mother, and believe that much evil might be averted were the contents generally and carefully studied. We wish that space were afforded us to present an analysis of it to our readers. We must content ourselves, however, with a hearty commendation of it to them, with the conviction that they cannot fail to derive benefit from its teaching, and from the extended experience of its author.

*Medical Jurisprudence.* By ALFRED TAYLOR, M. D., F. R. S., &c., &c. *Third American, from the Fourth London edition.* Edited, with additions, by ED. HARTSHORNE, M. D., &c., &c. Philada. Blanchard & Lea. 1853.

The author's belief, that this volume has already found a wide circulation, not only among his own profession, but gentlemen practising at the bar, is a well-founded one. Certain it is, that no similar book, with the exception of Beck's, has been more popular in this country. Dr. Taylor has displayed great diligence and fidelity in accumulating all the valuable matter presented by journals and other sources, since the previous edition, while the American editor has introduced all the important facts and cases that have occurred in this country. The present edition, therefore, we think, fully represents the existing state of knowledge both abroad and at home.

---

*Principles of Medicine: comprising General Pathology and Therapeutics, and a brief general view of Etiology, Nosology, Semeiology, Diagnosis, Prognosis, and Hygienia.* Edited, with additions, by M. CLYMER, M. D., &c. *Fourth American edition, revised.* Philada. Blanchard & Lea.

Another new edition of this popular, we may say now, standard work, evidences a desire on the part of the profession to become familiar with *principles*, and we know no work to which we can, with more safety, direct the student and practitioner than this. Although no new English edition has recently appeared, the American reprint has been carefully revised and posted by the editor, and much additional matter added. It should be on the shelves of every professional library.

---

*On the Nature and Treatment of the Deformities of the Human Frame: Being a Course of Lectures delivered at the Royal Orthopœdic Hospital, in* 1843. By W. J. LITTLE, M. D., Physician to the London Hospital, etc., etc. London. Longman, Brown, Green & Longmans. 1853. pp. 412.

We are indebted for this valuable monograph, certainly the most complete on the subject in the language, to the London publisher. The author considers it, as it is in some sense, a third edition of his *Treatise on the Nature and Treatment of Club-Foot*, published fourteen years since: and it consists in the main of a reprint of his Lectures on the subject, published originally in the London Lancet, in 1843. Dr. Little has, however, interwoven with the Lectures the results of his additional experience, and has adapted the work to "the altered position of the pathology and treatment of distortions at the present period, compared with its state when Stromeyer commenced his labors."

The subjects are, 1. Deformities in General; 2. Deformities from Wounds and Diseases of Joints, Accidents, Rheumatism, &c.; 3. Deformities from Spasm, Paralysis, Burns, Habitual Retention in One Position; 4. Deformities from Rickets, Weakness, and Curvatures of Bones; 5. Congenital Distortions, Club-Foot, Club-Hand, &c.; 6. Congenital Malformations, Monstrosities; 7. Distortions of the Spine.

This brief outline of the contents of this very useful and practical work, is perhaps a sufficient notice of it on the present occasion, as it has been already substantially for a considerable time before the profession. We shall offer our readers, however, a detailed extract from its pages, on a subject of recent interest, the treatment of anchylosis by forced extension, which is very judiciously treated by Dr. Little.

"The art of surgery has, however, recently received an invaluable addition to its means of usefulness, by the discovery of the anæsthetic properties of ether and chloroform; and orthopædic practitioners have not been slow in availing themselves of the assistance these means are calculated to render in the cure of deformities. Hence the employment of forcible extension with the aid of chloroform or ether. By chloroformisation, the two great obstacles to the employment of force adequate to straighten or bend a contracted limb,—namely, pain and voluntary muscular resistance,—are removed. As soon as these impediments disappear, the hands of the single operator, and his single mind, applied to the parts, encounter the physical resistance only of the deformed parts; comparatively gentle manipulations now acquaint him with the nature and amount of difficulty; he can feel his way in the application of greater force; feels and perceives the resistance of parts successively overcome, in an anatomical order; if greater rigidity still oppose, a few movements of the joint backwards and forwards prepare the way for a more extensive yielding; and often the practitioner has the satisfaction of being able to effect every natural movement of the joint, and satisfy himself that a cure is obtainable.

"Chloroformisation, with manipulations, and the use of a certain degree of force, is often of great service as a means of diagnosis; by it the practitioner is enabled to ascertain what proportion of the deformity is due to shortening of soft parts, and how much mischief the articular surfaces may have undergone. He may have reason to conclude, from the amount or nature of the resistance, and the change of articular surfaces, that restoration of the form and functions of the part is impracticable, and thus save much time, which without chloroform might have been uselessly expended in an attempt to straighten the part.

"After straightening or bending the limb, as the case may have required, by means of this forcible procedure, the part should be lightly secured in a retentive instrument or upon a common splint, adjusted so as to maintain the position more favorable than that which obtained before the operation, though not in the new position, *i. e.*, the entirely straight or bent position into which the hands of the surgeon may have brought it. For, as soon as the effect of the chloroform upon the sensorial system disappears, the patient will arouse to the conviction of the violence which may have been employed, the part may be acutely painful, and incapable of sustaining the pressure of a tight bandage or ligature.

"The author has usually contented himself with the increased knowledge of the nature of the case, with the satisfaction of knowing that the part could be improved in form and function, and that as the resting parts had once yielded, that they would afterwards oppose less resistance to cure; whether the means subsequently employed should be simple manipulations, the use of mechanical apparatus, or repeated administrations of chloroform, and 'forcible extension.' The surgeon who should attempt the forcible binding down of a long-deformed limb immediately after 'forcible extension,' would betray a lamentable ignorence of the pathological condition of the living parts in the immediate vicinity and within the deceased articulation. Although the muscular structures may have yielded under chloroform, and indurated fasciæ and old adhesions may have been overcome by tearing, it will be remembered, that much adaptation on the part of nerves, blood-vessels, and absorbents to the altered position of structures needs to be accomplished. The author has found that, by taking moderate means of retaining as much improvement after the forcible extension as can be borne by the sufferer, by unsparing use of lotions of spirits of wine, no inflammation of joints thus straightened has supervened. He has witnessed no untoward accident; and, by employing the ordinary means of gradual extension, and repeating once or twice the chloroform, and complete extension while the patient lay under its influence, he has restored cases, the relief of which would have required many months of ordinary treatment, or would otherwise have constituted very mediocre cures."

The work is illustrated by upwards of 160 well executed engravings and diagrams.

